# Induction of neutrophil apoptosis by a Bcl-2 inhibitor reduces particulate matter-induced lung inflammation

**DOI:** 10.18632/aging.101477

**Published:** 2018-06-26

**Authors:** Xinwei Geng, Xiaohui Wang, Man Luo, Meichun Xing, Yinfang Wu, Wen Li, Zhihua Chen, Huahao Shen, Songmin Ying

**Affiliations:** 1 Department of Pharmacology and Key Laboratory of Respiratory Disease of Zhejiang Province, Department of Respiratory and Critical Care Medicine, Second Affiliated Hospital, Institute of Respiratory Diseases, Zhejiang University School of Medicine, Hangzhou 310009, China; 2 Key Laboratory of Respiratory Disease of Zhejiang Province, Department of Respiratory and Critical Care Medicine, Second Affiliated Hospital, Institute of Respiratory Diseases, Zhejiang University School of Medicine, Hangzhou 310009, China; 3 State Key Laboratory of Respiratory Diseases, Guangzhou, Guangdong 510120, China; * Equal contribution

**Keywords:** particulate matter, lung inflammation, neutrophil, apoptosis, Bcl-2, ABT-199

## Abstract

Background. Environmental particulate matter exposure can cause various respiratory problems including aggravated asthma, decreased lung function and increased respiratory symptoms. However, the molecular mechanisms underlying PM-induced lung inflammation are incompletely understood. Effective therapeutic strategies are required.

Results. A mouse model of particulate matter-induced lung inflammation was used to identify the pathology and the molecular mechanisms for particulate matter-induced lung inflammation. The mouse model revealed that particulate matter induced neutrophil-dominated lung inflammation. Neutrophils derived from particulate matter-instilled mice showed decreased apoptosis and elevated Bcl-2 expression. Further studies in vav-Bcl-2 transgenic mice made it clear that Bcl-2 overexpression caused a marked increase in neutrophils in bronchoalveolar lavage fluid. Furthermore, we found that the Bcl-2 inhibitor ABT-199 reduced particulate matter-induced lung inflammation, and induced apoptosis of neutrophils in particulate matter-induced lung inflammation mice model.

Conclusions. Particulate matter-induced lung inflammation is mediated in part by inhibition of apoptosis of inflammatory cells. Bcl-2 is responsible for the reduced apoptosis of inflammatory cells in particulate matter-induced lung inflammation. The Bcl-2 selective inhibitor ABT-199 reduces particulate matter-induced lung inflammation by inducing the apoptosis of neutrophils and might be a promising drug for the treatment of particulate matter-induced lung inflammation.

## Introduction

Particulate matter (PM) causes various health problems and has been shown to be related with the incidence of respiratory diseases and the mortality of lung cancer [1]. Epidemiological evidence suggests that PM exposure can both induce acute inflammatory response and chronic lung inflammation [2].

Smaller particles are more harmful. They can reach deeper inside the respiratory tract. Studies have shown that particles with a diameter of about 10 μm are usually deposited on the upper respiratory tract. Particles with a diameter less than or equal to 2 μm usually enter the deeper parts of the lungs including the bronchioles and alveoli [3]. After these fine particles enter the body and reach the alveoli, they can directly affect the lung ventilation function resulting in poor ventilation or even hypoxia. Epidemiological evidence indicates that PM not only induces an acute inflammatory response, but also chronic lung inflammation. The risk of acute respiratory disease is significantly increased when the patient is in a high PM environment. Some chronic respiratory diseases may be induced at high PM concentrations over a long period of time [4].

One study found that PM exposure can induce airway hyperresponsiveness in mice and neutral granulocyte lung inflammation [2]; exposure to a certain concentration of PM environment will cause asthma flare-ups—especially neutrophils in asthma patients with significantly increased asthma symptoms [5].

Inducing inflammatory cell death and removing infiltrated inflammatory cells is an important strategy in the treatment of inflammatory diseases [6]. Thus, the development of a drug capable of inducing apoptosis or death of inflammatory cells such as an inhibitor against apoptosis-suppressing protein Bcl-2 is a promising treatment for the treatment of inflammatory diseases [7]. ABT-199 is a small molecule BH3 domain peptide mimetic originally used tumor treatment [8]. This inhibitor can selectively bind and antagonize the apoptosis-suppressing Bcl-2 family, which induces tumor cell death [9]. Previous studies have utilized ABT737/199 as the treatment for steroid-insensitive lung inflammation [6].

Here, we investigated the pathological feature of PM-induced lung inflammation and evaluated the essential role of Bcl-2 for the progress and persistence of PM-induced lung inflammation. Finally, we found that ABT199 attenuated PM-induced lung inflammation via inhibiting Bcl-2.

## RESULTS

### PM-induced lung inflammation is dominated by neutrophil accumulation, and PM reduced the apoptosis and death of inflammatory (neutrophil) cells in bronchoalveolar lavage fluid (BALF)

**Figure 1:**
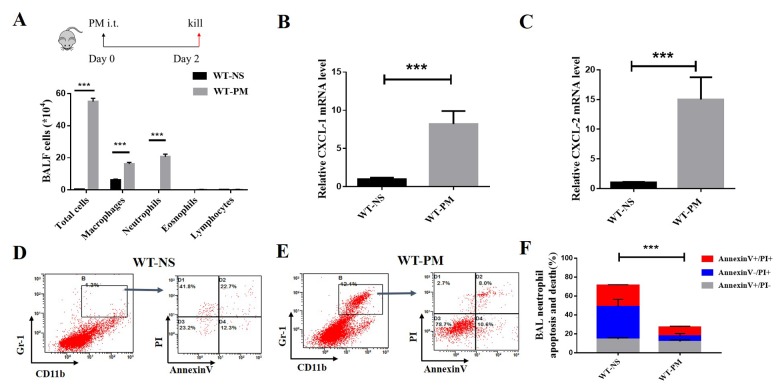
**PM-induced lung inflammation is dominated by neutrophil accumulation, and PM reduced the apoptosis of neutrophils in BALF.** We established a PM-induced lung inflammation model with instillation of PM at 100 μg/d/mouse for 2 days in WT mice (n=5 to 7 per group). PM increased the total number of macrophages, neutrophils, eosinophils, and lymphocytes in BALF. (**A**) Inflammatory cytokines such as the mouse chemokine (C-X-C motif) ligand 1 (CXCL-1) and CXCL-2 were significantly increased in WT-PM mice (**B** and **C**). Apoptosis in neutrophils were determined by Annexin V and PI staining based on the gating of Gr-1+/CD11b+ by flow cytometry. PM decreased the apoptosis of neutrophils in BALF cells (**D**-**F**).

We established a PM-induced lung inflammation mouse model by intratracheal instillation of PM suspension at 2 mg/ml for two days. This resulted in PM-induced, neutrophil-dominated lung inflammation. The results showed that the PM intervention group could significantly increase the total number of BALF cells compared with the NS (normal saline) group (P <0.05). The proportion and the absolute number of neutrophils both increased after PM treatment. The ratio of neutrophils in the alveolar lavage fluid of WT-PM mice was 32.36%, compared with 3.04% of neutrophils in WT-NS. This is about 10-fold higher than the WT-NS group (Fig. 1A).

We used Q-PCR to quantify the inflammatory cytokines such as mouse chemokine (C-X-C motif) ligand 1 (CXCL-1) and mouse chemokine (C-X-C motif) ligand 2 (CXCL-2) in the lung tissue. We found that PM significantly increased the expression of CXCL-1 and CXCL-2 (Fig. 1B and Fig 1C). Next, we detected the apoptosis and cell death of inflammatory cells in the total BALF cells. The apoptosis level of neutrophils was determined by Annexin V and PI staining based on the gating of Gr-1+/CD11b+ by flow cytometry. The percentage of neutrophil apoptotic cells in WT-NS is about 40%, which is twice as high in WT-PM (18.6%; Fig. 1 D-F). These results show that the PM induced obvious neutrophils-dominated lung inflammation, reduced the apoptosis and cell death of neutrophils in BALF cells, and increase the survival of inflammatory cells.

### PM increased the expression of anti-apoptosis protein Bcl-2 in BALF inflammatory cells

**Figure 2:**
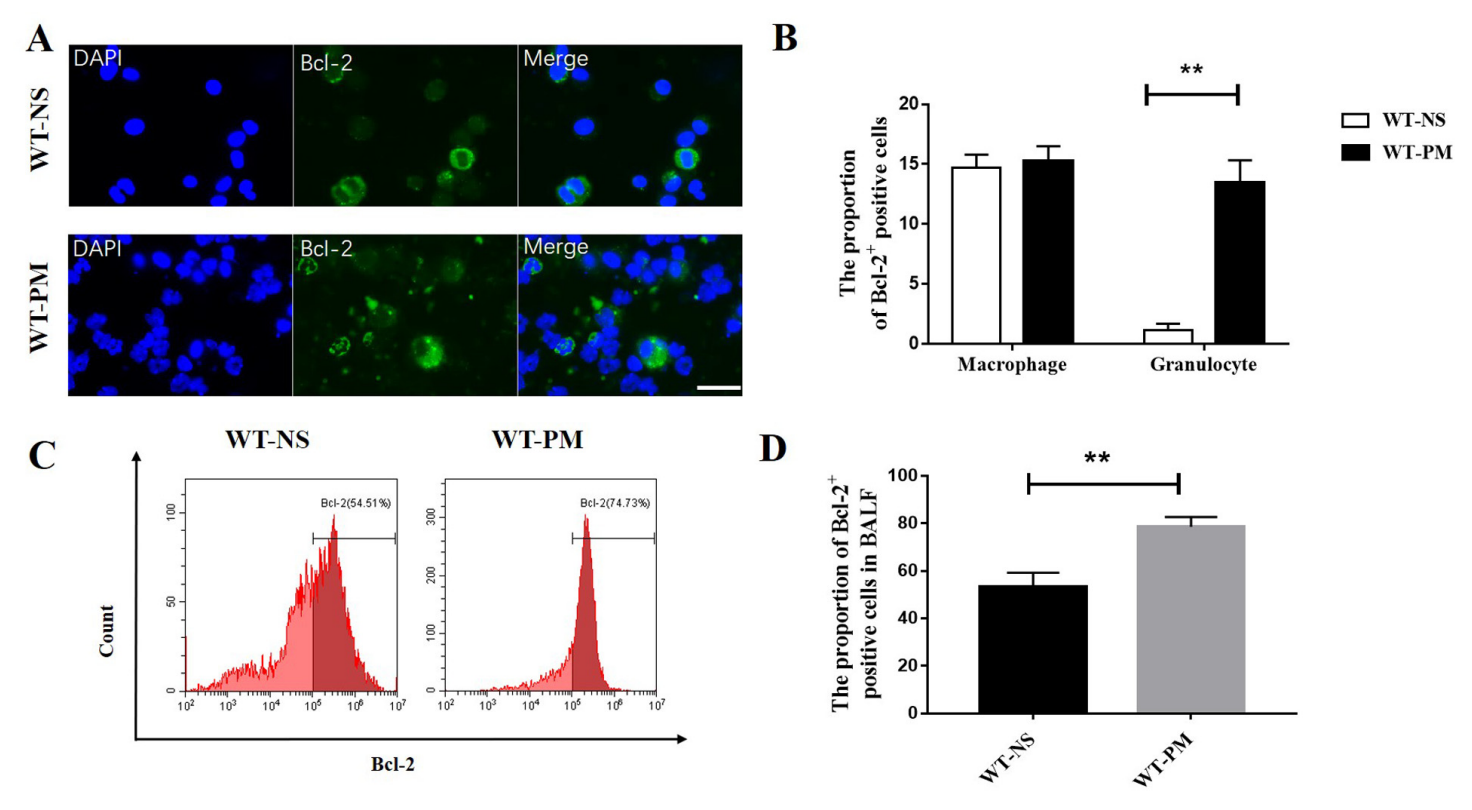
**PM increased the expression of Bcl-2 in BALF inflammatory cells.** We counted the Bcl-2 positive cells in BALF cells by immunofluorescence assays between WT-NS mice and WT-PM mice (scale bar=20 μm). Cells with segmented nucleus were considered as granulocytes. Data are mean/SEM from 5 to 7 independent experiments, n>200 (**A** and **B**). BALF cells were isolated from WT-PM mice, and intracellular Bcl-2 expression was assessed by flow cytometry. The percentage of Bcl-2-positive cells is higher than WT-NS in BALF (**C** and **D**).

We detected the percentage of Bcl-2 positive cells in total BALF cells by flow cytometry. The percentage of Bcl-2 positive cells in the total BALF cells in the PM group was 73.51%, which is higher than the NS group (54.71%; Fig. 2C-D). We also investigated the expression of Bcl-2 in different inflammatory cells and found that there was no difference in the percentage of Bcl-2 positive cells in the macrophages between PM and NS group, but the percentage of Bcl-2 positive cells in granulocytes was significantly increased after PM instillation (Fig. 2A-B).

### PM induced more serious lung inflammation in vav-Bcl-2 transgenic mice than WT mice

To further verify the role of Bcl-2 in PM-induced lung inflammation, we next constructed a PM-induced lung inflammation using the vav-Bcl-2 transgenic mice (Bcl-2 overexpression under the control of panhematopoietic *Vav* promoter). We analyzed relevant inflammation indexes at 24 hours, 48 hours, and 96 hours after the last PM instillation in both wild-type and vav-Bcl-2 transgenic mice. The total number of BALF and the differential counts of inflammatory cells were analyzed at 24 hours, 48 hours, or 96 hours after last PM instillation.

**Figure 3:**
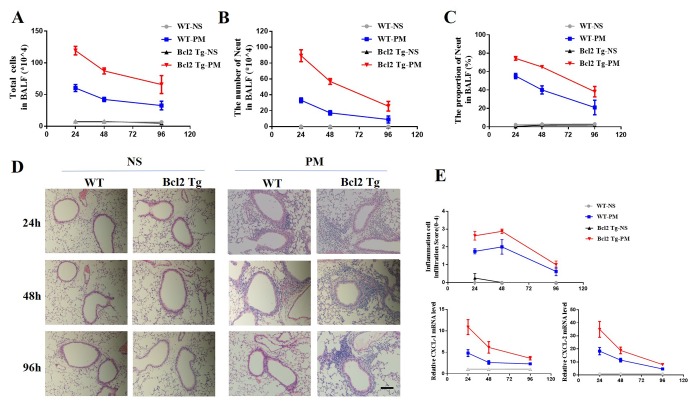
**Delayed resolution of PM-induced lung inflammation in vav-Bcl-2 transgenic mice.** To further examine the role of Bcl-2 in regulation of lung inflammation in vivo, vav-Bcl-2 transgenic mice (Bcl-2 overexpressing mice) were instilled with PM at 100 μg/d/mouse for 2 days (n=5 to 7 per group). This resulted in marked accumulation of total inflammatory cells and neutrophils in the bronchoalveolar lavage fluid (BALF). Both of these were significantly increased in the vav-Bcl-2 transgenic mice (**A**-**C**) after 24 h, 48 h, and 96 h instillation of PM. Histochemical staining (HE) identified inflammatory cell recruitment (scale bar=100 μm). Elevated levels of inflammatory cells were recruited in bcl-2 overexpressed mice. There was delayed resolution of inflammation in bcl-2 overexpressing mice (**D** and **E**). The expression of CXCL-1 and CXCL-2 were also analyzed at 24, 48 and 96 h after instillation with PM solution (**E**).

The total BALF number and the total number of neutrophils in BALF in vav-Bcl-2 transgenic mice is more than in wild-type mice. This indicated that the Bcl-2 overexpressing mice were more sensitive to particulate matter than wild-type mice. The PM-induced lung inflammation is an acute inflammation that subsidizes over time. We found that the speed of inflammation resolution is slower in Bcl-2 overexpressing mice than wild-type mice (Fig. 3A-C).

Next, we used hematoxylin and eosin (H&E) staining to demonstrate the recruitment of inflammatory cells. The H&E staining showed that the Bcl-2 overexpressing mice had increased inflammatory cell recruitment in the surrounding airways (Fig. 3D-E). These results suggest that over-expression of Bcl-2 was detrimental to PM-induced lung inflammation.

We also detected the mRNA expression of inflammatory cytokines CXCL-1 and CXCL-2 as well as the expression of CXCL-1 and CXCL-2 in the lung tissue of vav-Bcl-2 transgenic mice. These were obviously higher than the wild-type mice (Fig. 3E). At all three time points (24 h 48 h and 96 h) after PM instillation, the Bcl-2 transgenic mice expressed more CXCL-1 and CXCL-2. The Bcl-2 overexpressing mice showed more serious and durable inflammation than WT mice (Fig. 3E).

### Bcl-2 inhibitor ABT-199 reduced PM-induced lung inflammation by inducing neutrophil apoptosis

**Figure 4:**
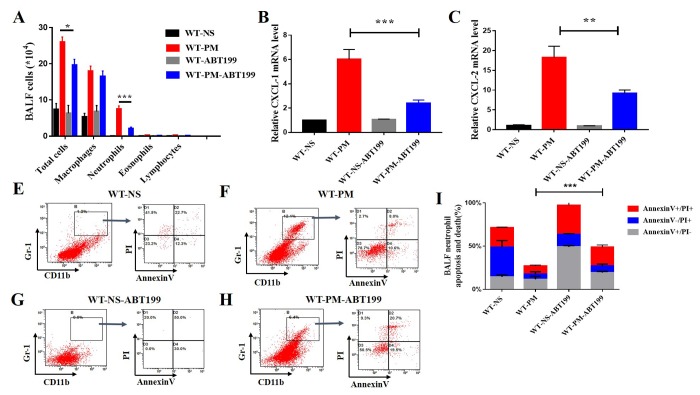
**ABT-199 alleviates PM-induced lung inflammation.** After instillation of ABT-199 in the PM inflammatory model, the total BALF cells and the number of neutrophils were significantly decreased (n=5 to 7 per group) (**A**). After instillation of PM, ABT-199 also decreased the expression of CXCL-1, CXCL-2 in lung tissue (**B** and **C**). Apoptosis levels were determined by Annexin V and PI staining based on gating of Gr-1+/CD11b+ by flow cytometry. ABT-199 induced apoptosis of Neutrophils in BALF cells in different groups (**E**-**I**).

Particulate matter induced apparent lung inflammation and high anti-apoptotic protein Bcl-2 expression in the inflammatory cells. Thus, we next applied ABT-199 for the treatment of PM-induced lung inflammation. After instillation of 2 mg/ml PM solution for two days, the mice were treated with Bcl-2 selective inhibitor ABT-199. The BALF were collected, and the BALF cells were counted with a differential. We found that the total cell number of BALF in ABT-199-PM treated group is 19.67×10^4^, less than 26.24×10^4^ in NS-PM treatment group (Fig. 4A). Versus the NS-PM treatment group, the proportion of neutrophils in BALF is also decreased after treatment with ABT-199 (11.92% vs 30.72%) (Data not show).

Next, we detected the mRNA expression level of inflammatory cytokines such as the mouse chemokine (C-X-C motif) ligand 1 (CXCL-1) and CXCL-2 in the lung tissue after treatment with Bcl-2 inhibitor ABT-199. The results indicated that ABT-199 decreased the expression of inflammatory cytokines CXCL-1 and CXCL-2 in the lung tissue of PM-induced lung inflammation (Fig.4B and C). The residual cytokines might be secreted by apoptotic alveolar cells as reported before [10]. The ABT-199 not only decreased the number of inflammatory cells in BALF but also reduced the secretion of inflammatory factors in the PM-induced lung inflammation model. These results indicate that ABT-199 can significantly alleviate the PM-induced inflammation. However, it remains unclear how ABT-199 relieves PM-induced lung inflammation.

To solve this problem, we detected the apoptotic level of inflammatory cells after treatment with ABT-199. We established the PM-induced lung inflammation model and then treated with ABT-199 at 24 hours after the PM instillation to relieve inflammation. We collected the total BALF cells from different treatment groups. The apoptotic and cell death from neutrophils were determined by Annexin V and PI staining based on flow cytometry gating of Gr-1+/CD11b+. The percentage of dead apoptotic neutrophils in the ABT-199 treatment group is about 48.73%, which is more than 27.13% of the NS-PM group (Fig. 4E-I). These data show that ABT-199 increases the apoptosis of inflammatory cells in PM-induced lung inflammation model, which suggests that ABT-199 relieves the PM-induced lung inflammation by inducing the apoptosis of inflammatory cells.

## DISCUSSION

Previous studies have showed that PM can induce various respiratory diseases [2]. We found that PM resulted in acute lung inflammation and recruited neutrophils to the airway of our PM mice model. While expression of Bcl-2 protein did not increase in the lung tissue of the lung inflammation model after PM instillation, the BALF neutrophils showed elevated Bcl-2 expression. To further reveal the role of Bcl-2 in PM-induced lung inflammation, we employed Bcl-2 transgenic mice in our PM-induced lung inflammation model. The results showed that PM-induced more serious and longer inflammation in the Bcl-2 transgenic mice.

ABT-199 is a successful Bcl-2 inhibitor and is under clinical trials for various diseases. Tian et al. showed that ABT-199 could be a potential treatment for steroid-insensitive airway inflammation [6]. Thus, we next utilized the Bcl-2 selective inhibitor ABT-199 to treat the PM-induced lung inflammation model and found that ABT-199 could promote the apoptosis of neutrophils and relive PM-induced lung inflammation.

Respiratory diseases are common including asthma, bronchitis, pneumonia, etc. They decrease quality-of-life [11]. Respiratory diseases are affected by many factors, and the incidence is closely related to the degree of environmental pollution and the climatic conditions. Studies have shown that the incidence of respiratory diseases increase significantly when air pollution increases [12]. Atmospheric particulate matter is an important part of environmental pollution. The PM index in a region's air is often used to evaluate the degree of environmental pollution.

PM are usually present in the atmosphere in the form of suspended matter or aerosols [13] and can grouped by size. Coarse particles are less than 10 microns in diameter (PM 10), fine aerosols are less than 2.5 μm (PM 2.5), and ultrafine particles have aerodynamic diameters below 0.1 μm (PM 0.1) [14]. Because of the relatively larger specific surface area of PM2.5, it can carry more harmful substances. This increases its impact on human health.

Although we found elevated expression of Bcl-2 in the PM-induced lung inflammation models, the mechanism underlying increased expression of anti-apoptosis protein Bcl-2 in inflammatory cells after PM instillation is unclear [15]. Apoptosis is characterized morphologically by chromatin condensation, genomic DNA fragmentation, and cell membrane dissociation [16]. Bcl-2 was first discovered because of its involvement in B-cell malignancies where chromosomal translocations activate the gene in about 80–90% of follicular non-Hodgkin’s lymphomas [17]. The Bcl-2 protein is an intracellular protein that inhibits apoptotic cell death induced by various stimuli in different cell types [15].

Air pollution is also associated with the lung cancer development [18]. Drugs targeting Bcl-2 family proteins have been tested in clinical trials in hematological malignancies. They seem promising, with the most significant drawbacks including gastrointestinal toxicity and thrombocytopenia [19]. Newly developed ABT-199 shows high efficacy and reduced toxicities [20]. The application of apoptosis inducing agents like ABT-199 might be a promising strategy for lung cancer therapy.

In summary, we successfully constructed a short-term PM-induced lung inflammation model. The in vivo results showed that the counts of inflammatory cells increased. This improved the viability of inflammatory cells, which might be due to elevated expression of anti-apoptosis protein Bcl-2 in the inflammatory cells. We also found that Bcl-2 overexpressing mice had more inflammatory cells, which reduced its capacity for self-cleaning of inflammatory cells after instillation with PM. Finally, we used local administration of the selective Bcl-2 inhibitor ABT-199 in the airway and found that ABT-199 could effectively increase the apoptosis of inflammatory cells to alleviate PM-induced lung inflammation. These results indicate that ABT-199 would be a promising therapeutic target for the treatment of environmentally-induced lung inflammation.

## MATERIALS AND METHODS

### Ethics approval

The mice study was conducted in the agreement with the Experimental animal welfare and ethics committee of Zhejiang University.

### Mice

Male C57BL/6 mice (wild-type, aged 6-8 weeks) were purchased from the Animal Center of Zhejiang University and housed in a conventional animal facility. Vav-Bcl-2 transgenic mice (male, aged 6-8 weeks) were a generous gift from Professor Andreas Strasser (The Walter And Eliza Hall Institute of Medical Research) [6]. All animals were feed with standard pellet food and acidified water and housed in polypropylene cages, temperature and humidity were controlled at 22 ± 1 °C and 55% ± 5 respectively. The light was controlled with 12 h light/12 h dark cycle. The study was conducted in the agreement with the Experimental animal welfare and ethics committee of Zhejiang University.

### PM-induced lung inflammation

The PM used in this study is Standard Reference Material (SRM) 1649b. SRM 1649b was purchased from the National Institutes of Technology (Gaithersburg, MD, USA) [21]. PM was suspended and sonicated in sterile saline to a final concentration at 2 mg/ml. The mouse model of acute lung inflammation used mice treated with 100 μg PM (in 50 μl saline) per day for 2 days by intra-tracheal instillation [22].

### BALF collection and analysis

Twenty-four hours after the last exposure to PM, BALF was obtained with three instillations, each instillation was performed with 0.4 ml PBS injected into the lungs. These were drawn to collect the cells. The total number of BALF cells was counted, and then the remaining BALF was centrifuged at 400 g for 10 min at 4 °C. The supernatant was stored at - 80 °C and used for analysis of cytokines. The cell pellet was suspended in 200 μl PBS, and 10 μl of the suspension was spun onto glass microscope slides. Cells were stained with Wright–Giemsa stain (Baso, BA-4017), and the numbers of eosinophils, neutrophils, lymphocytes and macrophages were counted and classified under a microscope by counting over 200 cells.

### Histologic analyses

After treatment with PM, the lungs were collected and pretreated with formalin for 24 h. Then the lungs were embedded in paraffin. Sections were stained with hematoxylin and eosin following standard protocols [23]. Inflammation score was determined according to published guidelines [23].

### ABT-199 solution

ABT-199 is a selective Bcl-2 inhibitor. It is insoluble in water. The ABT-199 (Selleck, Houston, Tex) solutions were formulated in 30% propylene glycol, 5% Tween-80, and 65% D5W (5% dextrose in water, pH 4.2). The dose we used here is 100 μg/mouse with a concentration of 2 μg/μl.

### RNA isolation and quantitative real-time PCR analysis

RNA was extracted from lung tissue homogenates using Trizol (Invitrogen, 15596 026). cDNA was amplified using Reverse Transcription Reagents (Takara Biotechnology, DRR037A). The expression of CXCL-1 and CXCL-2 were measured by Q-PCR using SYBR Green Master Mix (Takara Biotechnology, DRR041A). Primers used for *CXCL-1* and *CXCL-2* Q-PCR were listed below (Table 1):

**Table 1:** Primers used for quantitative real time PCR analysis.

Species	Genes	Primer sequence	
Mouse	*CXCL-1*	Reverse	5’-CAGGGTCAAGGCAAGCCTC-3’
		Forward	5’-CTGGGATTCACCTCAAGAACATC-3’
Mouse	*CXCL-2*	Reverse	5’-CTCAGACAGCGAGGCACATC-3’
		Forward	5’-TGTCCCTCAACGGAAGAACC-3’

### Flow cytometric analysis of BAL cells

One day after the last exposure to PM, BALF cells were obtained with three 0.4 ml PBS washes injected into the lungs and then withdrawn to collect the cells. The total number of BALF cells were counted, and then the remaining BALF cells were centrifuged at 400 g for 10 min at 4 °C. The supernatant was stored at - 80 °C and used for analysis of cytokines. The cell pellet was suspended in 200 μl PBS and stained with Gr-1 (PE-Cy7, cat. 25-5931-81, eBioscience), CD11b (BV421, cat. 101236, Biolegend), Annexin V (MultiSciences, Hangzhou, China), and PI (MultiSciences, Hangzhou, China) for 30 minutes at 4 °C. Bcl-2 antibody was purchased from Santa Cruz Biotechnology (Dallas, US). Data were acquired with a FACScalibur flow cytometer and analyzed with FlowJo software (version 7.6, San Carlos, CA)

### Statistical analysis

Results are presented as means with SEM. Data were analyzed with GraphPad Prism 7 (GraphPad software, San Diego, CA). Differences between two groups were evaluated with Student’s t test. A value of P less than 0.05 was considered statistically significant.

### Availability of data and material

All data generated during this study are included in this published article.
